# On the Applications of Electricity to Medical and Surgical Therapeutics

**Published:** 1861-03

**Authors:** 


ART. IV.—1. *Traité des Applications de l'Électricité a la Thérapeutique Médicale et Chirurgicale.* Par A. BECQUEREL, Médecin de l'Hôpital de la Pitié, etc. Deuxième Édition. 8vo., pp. 550. Paris, 1860.

*Treatise on the Applications of Electricity to Medical and Surgical Therapeutics.* By A. BECQUEREL, Physician to the Hôpital La Pitié, etc. 2d edition, etc.

2. *De la Galvanisation par Influence appliqués au Traitement des Déviations de la Colonne Vertébrale, des Maladies de la Poitrine, des Abaissements de l'Utérus,* etc. Par le DOCTEUR J. SEILER. 8vo., pp. 159. Paris, 1860.

*On Galvanization by Influence applied to the Treatment of Deviations of the Vertebral Column, Diseases of the Chest, Prolapsus Uteri,* etc. By DR. J. SEILER. 8vo., etc.

3. *Electro-Physiology and Electro-Therapeutics; showing the best Methods for the Medical Uses of Electricity.* By ALFRED C. GARRATT, M.D., Fellow of the Mass. Med. Society. 8vo., pp. 708. Boston, 1860.

THE applications of electricity to the uses of the physician form a prominent feature in the more modern treatises on therapeutics. Like most remedial agents which have received popular attention, this potent stimulant deserves a large share of the consideration which has been bestowed upon it, but has been pushed by its enthusiastic admirers occasionally farther than its merits or its utility would justify. We thus see it called upon, like some of its older associates in the list of the materia medica, to do active service in the cause of therapeutics, under a thousand varied circumstances, when rational philosophy and sound reasoning would teach the advocates of its employment that its powers should be limited to fields from which it cannot be abstracted with any probability of success. We are quite sure that some of the applications of electricity, in whatever form it may be employed, which works on that subject recommend, are but suggestions for its use, which have not been, and probably cannot be, carried out with benefit to the patient or with satisfaction to the practitioner.

A few of them perhaps are founded upon the enthusiasm of ardent advocates of its utility, while others seem to be suggested by a reasoning from analogy that because this peculiar mode of medication is applicable in one class of cases, it may be safely and successfully resorted to in those which appear to have a similarity of pathological phenomena. Nevertheless, the uses of electricity are so numerous, and its modes of application so varied, that these very suggestions, though often founded on erroneous premises, may, by opening out a new field of thought for the practitioner, lead at last to conclusions which may be of practical utility to mankind, by increasing the domain of curative medicine, and by relieving conditions of otherwise helpless prostration.

We have been led to these thoughts by a glance at the number of works on this one branch of medical science or art—whichever we may call it—with all its ramifications, which have appeared within the last few years, emanating from the pens of European and American writers. The three works whose titles head this article are perhaps among the latest of those issued in France and America. The treatise of Dr. Seiler, unlike those of Drs. Becquerel and Garratt, is devoted to the consideration of a few special morbid conditions, and the application of a special form of electrical influence to their treatment. The other publications contain all that it was practicable to obtain upon peculiarities of electrical instruments, and the conditions under which they may be made subservient to worthy uses in the hands of the physician. Let us first examine as a novelty the mode in which Dr. Seiler employs the powerful agent for good or evil which chemical science long since bestowed upon the persevering inquiries of the philosopher.

In 1853 the author undertook a series of experiments with the view of discovering, if possible, a means of producing local insensibility. Among other experiments, he employed the currents of electro-galvanic influence, after the manner of magnetizers, by making passes, with the excitors, over particular portions of the body, through the clothing, and without touching the skin of the patient, so that there was no discharge of the electric spark. But these experiments being unattended with satisfactory results, others were instituted, and he found in one instance, a person recognized subsequently as having an extraordinary muscular excitability, that the arms became numb, the muscles contracted, and there was great difficulty in moving the fingers. There was no absence of sensibility. By further experiments and by modifications of the apparatus, he found it possible to produce in almost every case tolerably powerful muscular contractions or rigidity, which lasted several hours.

The peculiar apparatus, which in Dr. Seiler's hands has been attended with the most satisfactory results, is minutely described in his work. The details of its construction might, if critically examined, possess some interest to the chemist, but our limits prevent us from giving them at length. The liquids used are concentrated nitric acid, hydrochloric acid, and distilled water; the constancy of action being given to the battery, as Dr. Seiler thinks, by the addition of the hydrochloric acid.

The sensations experienced by the patient to whom the battery is applied, are at first those of elevation of temperature, extending along the surface and deeply into organs, without the slightest appearance of redness externally; a sedative action on the system, calming all pain, at least momentarily, as evinced by its power to relieve toothaches and neuralgias; and a sense of increased vigor, as if it had exerted a decided tonic action also; gradual contractions of the muscles, increasing in intensity, and being succeeded, if long continued, by vertigo, headache, fatigue, etc. In some of the more sensitive cases submitted to his experiments, Dr. Seiler produced what he calls a localized cataleptic attack, (*catalepsie localizée*,) and he was induced to extend the application of the results at which he had arrived, to the treatment of certain morbid conditions, from his observation that it was merely necessary to change the places of application to obtain very uniform contractions in the different muscles of a part. It was this fact which led him to employ this mode of treatment in deviations of the vertebral column. In his enthusiasm, he appeals to its tonic agency to strengthen the nervous system, give vigor to the movements, and good spirits to cheer the consumptive.

The special morbid conditions in which Dr. Seiler deems his apparatus applicable as a means of relief are very diversified in their nature and pathology, as deviations of the spinal column, phthisis, (by producing dilatation of the chest,) laryngitis, asthmatic affections, prolapsus, etc. of the uterus, and some cases of neuralgia. After referring to the value of spirometry as a "most precious means of diagnosis," a not infallible one, physiologists tell us, he remarks :—

"Persons whose conformation of body presents all the signs of a tuberculous diathesis, or who are already subjects of that disease, carry themselves badly and stoop while walking, or sink down as soon as they are seated, from a relaxation of the tone of their muscles. With such cases I commence by exciting a strong rigidity in the upper part of the back to give afterward to the muscles of inspiration of the anterior surface of the thorax a point of support sufficient for their action. After having straightened the back, I pass to the chest; I make the patient draw a deep inspiration, and attempt to teach him to keep himself in this condition and to carry on his respiration only with the abdominal muscles and the diaphragm, while I excite a sufficiently strong rigidity. This alone maintains the chest in a state of dilatation for several hours, according to the degree of muscular excitability of the subject and the intensity of the current which has been employed. This rigidity diminishes a little the respiratory movement of the thoracic parietes, but there does not result from it any dyspnœa, because the diaphragm, which seems in some way to share in the galvanic excitement, contracts more in its turn and operates more energetically.

"We may at least be convinced in determining as well as possible the lowest limit of respiration at the base of the lung, that after an energetic galvanization this limit becomes from one to two finger breadths lower. Another fact which I have often had occasion to repeat and verify, and which confirms me in my opinion as to the part which the diaphragm bears in these cases is the following: non-febrile consumptives, when suffering from extreme oppression, find it promptly relieved on applying the excitors of the inductive current to the base of the thorax and to the epigastrium, while if the upper part of the chest is galvanized, this relief is not usually felt until toward the end of the application."

All portions of the chest are not equally dilatable; and on this point, also, we have the authority of Dr. Seiler's experiments.

"The most difficult part to dilate is the region under the collar-bone. We are constantly obliged to direct the patients to inflate this portion well, and it is necessary, as much as possible, to produce there the greatest rigidity. If this point is neglected, we run the risk of dilating only the lower part of the thorax, and the vital capacity of the lung is not increased in proportion.

"This dilatation, once obtained, is preserved for years, possibly until the time of commencing decrepitude; and I could mention a large number of cases of dilatation which I effected several years ago, with a success which has lasted to the present time."

A curious condition induced by the use of electricity by influence, in the hands of Dr. Seiler, is also described by him; but much of the description seems to be dictated by enthusiastic zeal, which may have magnified the results obtained. If, however, electricity has any such power over the respiratory apparatus, as Dr. Seiler supposes, his experiments may be well worthy of repetition.

"I must mention here an effect produced upon the very tissue of the lung by electricity by induction, an effect which almost all the persons I have treated have described to me. The greater part of these patients appear to experience, not only upon the skin or on the thoracic walls, but in *the very interior of the lung itself*, the sensation of a soft and agreeable warmth, which, according to them, calms the irritation which excites their cough; this sensation is followed by a feeling of comfort and vital force which relieves the suffering so frequently accompanying the oppression, and this oppression becomes weaker or disappears entirely, because inspiration has become freer. The same patients reply almost unanimously when they are questioned as to details, that before the galvanization they felt in the interior of the lung an obstacle which prevented them from taking a deep breath, which seemed to them to stop about the middle of the chest. There are even patients who imagine that they feel a kind of spasm (?) like a valve, which shuts and stops the inspiratory movement.

"As soon as the galvanism produces its effect, and this sensation of warmth penetrates into the interior of the organ, they experience a dilatation *of the pulmonary substance;* at the same time the spasm, or, as they prefer to call it, the obstacle, which has prevented free inspirations, gives way; the inspirations are deep and the oppression disappears completely, or at least the respiration becomes as easy as the actual state of the lungs renders possible."

How shall we explain this effect of electricity, an effect which, he urges, is not accidental, for he has observed and verified it under all possible forms? The explanation is ingenious, and probably correct; it is the one which presents itself most naturally to the experimenter.

"I admit that in a great number of consumptives the contractile fibres of the minute bronchial ramifications are more or less relaxed, and that the elastic fibres of the pulmonary vesicles lose a part of their elasticity. The expiratory movement remaining then almost entirely confined to the abdominal and intercostal muscles, the expiration becomes imperfect; the vital capacity of the lung is consequently diminished, and the air is only partially renewed within it. Electricity restoring to the contracting fibres their action, and to the elastic fibres their elasticity, facilitates expiration, and naturally renders the new inspiration easier, fuller, and more prolonged."

The remainder of the work is filled with cases and experiments exhibiting a perseverance in the employment of this particular mode of treatment, which, to say the least of it, is worthy of the success which has attended Dr. Seiler's efforts. We have referred mainly to the influence of electrical applications in affections of the chest, because that subject forms so prominent a feature in the work before us. In certain morbid conditions of the uterus, in various forms of neuralgia, and even in two cases of subluxation of the knee, Dr. Seiler found some plausible reason for the use—and, according to him, the successful use—of his remedy. We have devoted considerable space to his experiments and conclusions, because we have thought there was some novelty in his application of electricity, and a great deal of commendable zeal displayed in its adaptation to the relief of certain morbid conditions.

We pass to the consideration of the comprehensive volume of a chemist, physiologist, and pathologist, whose treatise on the application of electricity to medical and surgical therapeutics contains a large amount of useful information on points which have importance alike to physician, surgeon, and the man of general scientific knowledge. The name of the author, occupying as he does the posts of Physician to the Hôpital de la Pitié, and "Professeur agrégé" of the Faculty of Medicine of Paris, is a guarantee that the authority for the facts mentioned in the volume before us is reliable.

The work embraces a far larger number of medical applications of electricity than any we remember to have seen. One peculiarity of the treatise will meet with the cordial approval of many members of the profession, who might otherwise have some difficulty in comprehending in what special forms of disease this stimulant may be appropriately employed. We refer to the numerous subdivisions of paralysis, and the mode in which electricity may be beneficially used in each. In fact, while suggesting the *modus operandi* of special modifications of apparatus in different forms of that affection, it becomes to a certain extent necessary to refer to the pathology of the cases, and thus the author conveys lessons in pathology as well as in therapeutics. The divisions and subdivisions of paralysis constitute almost the whole range of affections in which electricity is employed therapeutically, and the great bulk of M. Becquerel's work is therefore occupied in the discussion of those subjects.

It was in 1857, we believe, that the author published a *Treatise on Electricity*, which was scarcely more than a collection of his clinical lectures at the Hôpital de la Pitié upon its medical relations. Its object was to check the almost universal application of this agent by those who did not belong to the medical profession, to cases in which it was not indicated, or was useless and injurious. M. Becquerel, in a proper spirit of consideration for the welfare of the suffering public, who to their other ailments sometimes incautiously add those which flow from the indiscriminate employment of a stimulant prescribed by ignorant charlatans, thinks the time has come when rules should be established for the methodical and rational use of electrization. He desires also to show in what a large number of cases its employment would be futile, or dangerous, or aggravate the affection the physician wishes to relieve.

M. Becquerel has, with praiseworthy zeal, repeated all the reported cures described in the medical journals, and now publishes the results, whether favorable or unfavorable, in the second edition, which we have before us. The volume, as the author himself says, is as much a critical work as an experimental *résumé*.

Before entering upon the discussion of the subjects embraced in the four general divisions of his treatise, the author gives, as an introductory chapter, an historical account of the medical applications of electricity, embraced in three periods, dating from its earliest employment to the present time. We are almost tempted to correct M. Becquerel's phraseology, and to suggest that the heading of the chapter should read, "*L'Histoire des Applications Françaises de l'Électricité,*" etc., for we look almost in vain for English names among the modifiers of apparatus, or for any that are not indubitably French.

Have the English done so little in regard to the remedial applications of electricity, that they deserve to have their claims wholly ignored in a scientific work professing to give a complete summary of the periods of progress in the use of a valuable remedial agent? We might mention several English works which are deserving of a place in his historical collection, and without which it is scarcely complete.

The history of the applications of electricity to medicine is divided into three very distinct periods.

"The *first* comprehends all that time during which use was made only of the electrical machine, of the Leyden jar, and of other apparatus based upon the application of the results of statical electricity. It extends to the commencement of this century. The earliest work upon this subject appears to have been one by J. Deshayes, of Orleans, in 1740, on the cure of hemiplegia by electricity, six years before the discovery of the Leyden jar.

"The *second*, that in which the galvanic battery, more especially the trough battery, and electro-puncture, were employed, comprises all steps taken from 1800 to 1831—the period of the discovery of the effects of induction.

"The *third* includes finally all that time during which interrupted currents and all the apparatuses of induction were made use of. It comprehends, consequently, the precise period during which the applications of electricity to the healing art have made their fullest development."

We translate from M. Becquerel's treatise the following sketch of the instructions transmitted to the Military Hospital of Algeria, in 1858, and published in the *Military Journal* for September 13th of that year, because it gives in a condensed form so much valuable information on the modes of employing electricity as a remedial agent.

These instructions contain directions for the establishment of electro-therapeutic treatment in different military hospitals, which are indicated. The diseases submitted to this treatment are to form a distinct division, and be under the direction of special physicians. The instructions give also an account of the various forms of apparatus employed in applying electricity; they describe in detail their *modus operandi*, and the care necessary to keep them in order. Then follow rules for the use of these machines. Finally, they furnish a list of the affections to which in the present state of the science the electrical treatment can be applied. It may be interesting to the reader to reproduce them here.

"The affections susceptible of being submitted to the action of electricity are embraced in the following catalogue:—

I—*Lesions of Mobility, or of the Movements.*—Depending on

1. Tumors developed on the track of the nerves, or in the cranium or vertebral column.
2. Cerebral hemorrhage.
3. Softening of the brain.
4. Lesions of the spinal column.
5. Traumatic injuries of the nerves.

II.—*Lesions of Sensibility.*

1. Anæsthesia.
2. Hyperæsthesia.
3. Perversions of nutrition.
4. Applications to surgery.
   - *a.* Aneurisms.
   - *b.* Erectile tumors.
   - *c.* Special cases for the use of the galvanic cautery."

Some of the theories advanced are disputed by M. Becquerel, and especially those which relate to neuralgia, atrophy, and several other affections.

Having thus added to his work a valuable historical summary of medical electricity, which, we may state, ends with the treatise of Althaus, published in London in 1859, the author divides the remainder of his book into three general parts and numerous subdivisions, as follows:—

Part I.—Administration of electricity by means of apparatus in which it accumulates in the static state, or by means of instruments which furnish electric currents.

Chap. I.—Employment of electricity accumulated in the static state, and furnished by ordinary machines.

Chap. II.—Employment of dynamic electricity.
Part II.—Mode of application of electricity to the organism, and its action upon the tissues.
Part III.—Applications of electricity to therapeutics.
Indications and contra-indications.
Chap. I.—Lesions of movements.
1. Paralysis of the muscles of life of relation.
2. Paralysis of the muscles of organic life.
Chap. II.—Lesions of the organs of the senses.
Chap. III.—Muscular atrophy.
Chap. IV.—Applications of electricity to various pathological conditions.
Chap. V.—Applications to surgical affections.
Chap. VI.—Dangers and inconveniences of the employment of electricity.

The indications and counter-indications to the administration of electricity in affections in which it may be employed are referred to.

These indications are:—

"1st. To re-establish the contracting power of muscles deprived of it. 2d. To re-establish general or special sensibility of the organs of the senses, destroyed or simply weakened. 3d. To restore to their normal condition contractility and sensibility, which have been exaggerated or perverted. 4th. To obtain a cutaneous revulsion, by producing a superficial pain and a capillary hyperæmia equally superficial and transient."

Besides these, there are a number of other affections, differing much in their natures, in which the currents may be advantageously called into requisition. These more modern applications of electricity may be referred to four special indications, which we here attempt to specify:—

"1st. To cauterize parts, and thrust some caustic agent, as a platinum wire heated to a white heat by means of electricity, into organs or deep parts, to which we could not venture to apply ordinary caustic; or, still better, to replace the sharp instrument by cautery, effected by means of the same platinum wire heated to white heat by electricity.

"2d. To employ electricity as a chemical agent; for example, in coagulating the blood in an aneurismal tumor.

"3d. To determine the destruction or resolution of tumors of different kinds, by causing electric currents to penetrate into their substance by means of special electric apparatus.

"4th. To modify the vitality of affected organs by acting on them by some agency or other, not yet clearly defined; a course only explicable in connection with the details of cases."

Any one who is at all familiar with the phenomena of electricity and its effects upon the system must be aware that certain conditions and peculiarities exist in individuals occasionally, which should be taken into consideration as difficulties in the way of the administration of this potent agent. These are, according to M. Becquerel:—

"1st. Nervous susceptibility, which may become a counter-indication to the administration of electric currents, and may force us to discontinue their use.

"2d. Electric idiosyncrasy. When this idiosyncrasy and electric susceptibility are developed, there is almost always a counter-indication to the use of this agent.

"3d. Old affections. It is important, when we are about to apply electricity to subjects who have been previously sufferers from such affections, to watch for the possibility of their return; and if this return occurs, or even threatens to occur, we must immediately renounce the use of electricity.

"4th. When an individual presents himself with an affection to which electricity would be applicable, but is also suffering from some other acute or chronic disease, it must be accepted as a rule that there is a counter-indication to the use of this agent. We must wait for some time after the disappearance of the disease before applying it.

"5th. Persistence of an organic lesion, which was the cause of the morbid phenomena, for the relief of which electricity has been used. Such cases are more frequent than is imagined, and constitute always a counter-indication to the use of electricity."

More than one hundred and seventy pages of the work are occupied in the discussion of the subject of Lesions of Movement, embracing loss or impairment of movement, to which is given the name of *Paralysis*, and the perversion or exaggeration of movement, which comprises *Convulsions and Contractions*. The author quotes largely from the experiments and observations of English authorities, especially from those of Dr. Marshall Hall. There is scarcely a morbid affection of the cerebro-spinal system on which paralysis may depend that does not receive from the able hands of M. Becquerel ample discussion on its pathology, treatment, etc. Then, too, we have a full investigation of those forms of paralysis which follow upon acute or chronic affections of the genito-urinary apparatus, and all the varieties of paralytic affections which seem to be symptomatic of the neuroses, or are associated with arthritic cases, or are indicative of general debility, or are symptomatic of some form of poisoning. Under this latter head we find diphtheritic paralysis, lead palsy, arsenical paralysis—indeed, all the forms of paralysis consequent upon the action of poisonous substances. The General Paralysis of the insane belongs to another division, which embraces also several other forms, difficult to classify.

In the work of Dr. Althaus,* the mode of action of electricity in

* Treatise on Medical Electricity, Theoretical and Practical, by J. Althaus, M.D. London, 1859.

paralysis was discussed so fully that M. Becquerel feels that he is doing him but justice in quoting the conclusions arrived at by him in answer to the question, *How is it that galvanism acts beneficially in paralytic diseases?* "Galvanism acts in paralysis," says Althaus, "by restoring the lost mobility to the molecules of the nerves and muscles, and by causing contraction of, and a more considerable supply of arterial blood to, the paralyzed muscles." This view the author considers hypothetical, and therefore unestablished.

The indications for treatment of paralysis by electricity, and the manner in which such treatment should be instituted, form an important feature in the work; but we must pass to other portions. Convulsions and contractions would seem to be affections which would contra-indicate the employment of an agent whose immediate effect is to produce an involuntary contraction, a decided convulsion of a special kind. Yet under certain conditions, and in certain varieties of convulsive action, special modes of application of galvanism are justifiable, as in those cases when the convulsions have continued longer than the primary cerebral and spinal lesions, which have been cured or have disappeared; or have persisted after relief from poisoning, as of strychnia, opium, etc.; or have constituted an important element of general neuroses, as hysteria, chorea, and epilepsy.

The author considers the best mode of prescribing electricity in some of these conditions to be by what he calls *médication hyposthénisante,* based on the fact that it is possible momentarily to deaden the normal mobility, or to paralyze the morbid contractility of a part, by particular modes of application of continuous or rapidly-intermittent currents. The author states the fact that this mode of medication has been attended with success, but we look in vain for an explanation of the precise *modus operandi* of its action, which gives it a superiority over other methods of applying electricity. Probably M. Becquerel, in this instance, may belong to the class of physicians to whom he refers in the latter part of the following sentence:—

"Les expérimentateurs ont employé les uns une méthode perturbatrice, les autres une véritable médication substitive, *un certain nombre, enfin, une méthode dont ils ne cherchent pas a expliquer les effets, mais qui guérit.*"

M. Becquerel, to make his work as complete as possible, gives it considerably greater bulk than it would otherwise possess, by recording the fruitless efforts of credulous physicians to extend the powers of electricity to the cure of diseases in which it is wholly inapplicable. In this way the work loses, to a slight extent, in conciseness what it gains, perhaps, in completeness. In a book, which has no index, but merely, after the French fashion, a table of contents, this fullness, if we may so call it, is scarcely a virtue; in such *richesse* there arises an *embarras* which a copious index would relieve.

We are glad to see so comprehensive an abstract of the contents of two works on mental alienation, and the applicability of electricity to its palliation or cure, because that subject is not fully discussed in works on insanity, and the two quoted are inaccessible to the general public through any other channel than that which M. Becquerel's work affords.

The remaining chapters of the work, which, in addition to other subjects, contain all that is novel in the surgical applications of electricity, do not need a special notice here. They are in keeping with the whole book, devoted to the scientific explanation of all the indications for its employment, and clear in the description of the modes of making it useful. We feel that we are doing but justice to each author, when we say that the works of M. Becquerel and Dr. Althaus are the best that have recently appeared in France and England upon the interesting points upon which they treat, and, we regret to say, no American publication yet issued is deserving of a place in the same rank of excellence.

The most recent cisatlantic treatise is that of Dr. Garratt, of Boston, a handsome octavo, which, in its general appearance, does infinite credit to its publisher. The construction of the work is simple, the aim evidently being to have as few grand divisions as possible, though this arrangement necessitates a crowding together of an immense number of items under each head. The great fault of the book is, we think, the attempt to produce a cyclopedia of electricity, rather than a rational account of electro-therapeutics, such as would be embraced in a treatise.

The consequence is that Dr. Garratt has inserted numerous facts and descriptions which are unnecessary to the elucidation of the subject. He has introduced into a work intended for the practitioner, the first principles of electricity, the description of the simplest forms of battery, etc., as if his book were adapted for the simplest tyro in medicine; and, although we have had abundant opportunity of appreciating the fact that on these elementary points in chemical knowledge medical men are soon apt to become rusty, it is scarcely in a work professing to teach the advanced portions of electrical theories and modes of practice that they would seek for the means of reviving their chemical attainments. The first thirty pages, for example, might be omitted entirely, and the vacuum caused by their omission filled by something more eminently medical.

We have been struck more than once in Dr. Garratt's volume with the similitude of the language to that of other writers, and we might be tempted to employ what he himself styles "unnecessary and detracting criticism," did we not feel that the author's preface was deserving of consideration in extenuation of it. The author deems it scarcely necessary to state, "except to forestall unnecessary and detracting criticism, that he has unavoidably employed the ideas often, as also the language of others." After a long attempt to prove how difficult it is, in quoting from other authorities, to avoid using their own language, he states that "where quotations are made, they are duly acknowledged." Why not always quote *verbatim*, then, instead of laying one's self open to the charge of plagiarism by imitating words and expressions?

Chapter II., on the Early History of Medical Electricity, would scarcely please M. Becquerel; the French are not there shown to be the only nation of chemical *savans* who have ever done anything to assist the cause of electricity. German and English names are quite as numerous, and deserve quite as prominent a position as their Gallic neighbors. We do not recall at this moment any *Alexander* Dumas, as distinguished for chemical achievements; yet Dr. Garratt gravely tells us that one of that name, probably the ubiquitous and popular writer of *Les Trois Mousquetaires,* had "in some instances succeeded in decomposing calculi in the living human bladder." Certainly a *novel* application of electrical currents!

A work adapted for practitioners, as before remarked, ought to take for granted that the chemical basis upon which electro-therapeutics is founded is already laid, and yet, in the next chapter, it has been thought advisable to go back to the simplest construction of instruments in order to exhibit how well the complicated forms may act, when properly employed. In this section of the work, especially, much might have been, and ought to have been omitted, without in the least detracting from its general usefulness. The galvanometer, for instance, (p. 121,) is an ingenious apparatus; but of how much advantage is it to electro-therapeutists?

With equal propriety might the illustrations be curtailed; many are introduced with no very ostensible purpose. Dr. Garratt seems to have been bent upon teaching almost every department of medicine to the student, while exhibiting the action and uses of electricity. One would think, in cursorily glancing at some portions of the work, that the binder had accidentally bound up portions of Wilson's Anatomy with the elementary parts of a work on chemistry, the subject of electricity from a treatise on materia medica and therapeutics, and chapters from works on surgery, obstetrics, and dentistry. Mere anatomical plates, with apparently no direct connection with the text, puzzle the reader with inquiries as to the reasons for their introduction. Some of the illustrations seem inappropriate, to say the least; we hope they are more useful than they appear.

Many portions of Dr. Garratt's work are well written and clear, and contain much valuable information on electro-physiology and electro-therapeutics. The great defect of the work we consider to be its length, seven hundred pages being occupied in the discussion of matters which, with proper pruning, might be reduced to at least half that amount. If the author would, before issuing a second edition, consider the inappropriateness of introducing mere elementary subjects in chemical science into his book, and omit a large portion of the general pathology of diseases, he would make it a much more useful and quite as effective a treatise on medical electricity. A physician is supposed to know something about the pathology of the cases he wishes to treat before applying for information as to modes of cure, and, if ignorant of pathology, would assuredly not seek to be enlightened in a work on electro-therapeutics.

Nevertheless, even with these defects, we have cause to thank Dr. Garratt for his industry in collecting facts, his simple classification of them, and his efforts to produce a useful systematic treatise on the medical and surgical uses of electricity. In the words of the author, the object has been "to confine himself to gleaning from the highest practical authorities, and the comparing of these with his own clinical experiences, then classifying and arranging the subject-matter so as to present the whole range of electro-therapeutic practice on a more systematic and scientific basis." In this end we think he might have been more successful, had he adhered to this purpose throughout his work, and not adopted, a few lines farther on, a somewhat different aim, "success in practice, not theory."

